# Association between work characteristics and epigenetic age acceleration: cross-sectional results from UK – Understanding Society study

**DOI:** 10.18632/aging.204327

**Published:** 2022-10-05

**Authors:** Anna Freni-Sterrantino, Giovanni Fiorito, Angelo d’Errico, Marianna Virtanen, Leena Ala-Mursula, Marjo-Riitta Järvelin, Paolo Vineis, Oliver Robinson

**Affiliations:** 1 MRC Centre for Environment and Health, Department of Epidemiology and Biostatistics, School of Public Health, Imperial College London, London W2 1PG, United Kingdom; 2 The Alan Turing Institute, John Dodson House, London NW1 2DB, United Kingdom; 3 Laboratory of Biostatistics, Department of Biomedical Sciences, University of Sassari, Sassari 07100, Italy; 4 Department of Epidemiology, Local Health Unit TO 3, Turin 10095, Italy; 5 School of Educational Sciences and Psychology, University of Eastern Finland, Joensuu FI-80101, Finland; 6 Division of Insurance Medicine, Department of Clinical Neuroscience, Karolinska Institutet, Stockholm 17177, Sweden; 7 Center for Life Course Health Research, Faculty of Medicine, University of Oulu, Oulu 90014, Finland; 8 Ageing Epidemiology (AGE) Research Unit, School of Public Health, Imperial College of London, London W2 1PG, United Kingdom

**Keywords:** epigenetic age, work characteristics, unemployment, job schedule

## Abstract

Occupation-related stress and work characteristics are possible determinants of social inequalities in epigenetic aging but have been little investigated. Here, we investigate the association of several work characteristics with epigenetic age acceleration (AA) biomarkers.

The study population included employed and unemployed men and women (*n* = 631) from the UK Understanding Society study. We evaluated the association of employment and work characteristics related to job type, job stability; job schedule; autonomy and influence at work; occupational physical activity; and feelings regarding the job with four epigenetic age acceleration biomarkers (Hannum, Horvath, PhenoAge, GrimAge) and pace of aging (DunedinPoAm, DunedinPACE).

We fitted linear regression models, unadjusted and adjusted for established risk factors, and found the following associations for unemployment (years of acceleration): HorvathAA (1.51, 95% CI 0.08, 2.95), GrimAgeAA (1.53, 95% CI 0.16, 2.90) and 3.21 years for PhenoAA (95% CI 0.89, 5.33). Job insecurity increased PhenoAA (1.83, 95% CI 0.003, 3.67), while working at night was associated with an increase of 2.12 years in GrimAgeAA (95% CI 0.69, 3.55). We found effects of unemployment to be stronger in men and effects of night shift work to be stronger in women.

These results provide evidence of associations between unemployment with accelerated ageing and suggest that insecure employment and night work may also increase age acceleration. Our findings have implications for policies relating to current changes in working conditions and highlight the utility of biological age biomarkers in studies in younger populations without long-term health information.

## INTRODUCTION

Assessment of biological age using biomarkers can provide an indication of overall health and risk of disease in later life, even in studies without long follow-up time. Epigenetic clocks are composite scores of DNA methylation levels at different CpG sites in the genome and are popular indicators of biological age. Multiple epigenetic clocks have been developed: The “first-generation clocks” of Horvath [1], developed from multiple tissues, and Hannum [2], developed from blood samples, were both trained against chronological age. The “second-generation” clocks, PhenoAge [3] and GrimAge [4], aimed to be a closer approximation of biological age through training using both biomarkers and mortality data. The second-generation clocks are more strongly associated with health and lifespan than the first-generation clocks [4]. Epigenetic age acceleration (EAA) is commonly defined as the differences between epigenetic clocks and chronological age and positive values of EAA indicate that an individual is experiencing accelerated aging (AA) and vice versa. EAA has been associated with several risk factors for non-communicable diseases, all-cause mortality, frailty, cardiovascular diseases [5], diabetes, cancer [1, 2], decline in cognitive ability, depression and anxiety [6, 7]. Most recently, the Dunedin Pace of Aging (DunedinPoAm) [8] was developed based on longitudinal biomarker and clinical data to measure the rate of biological ageing. Notably, DunedinPoAm, is more strongly associated with self-reported health than both the first- and second-generation epigenetic clocks [8]. An updated version of Dunedin pace of aging has been published in 2022 by Belsky et al. [9]. The newly released rate DunedinPACE incorporated additional follow-up clinical data and has been found to be associated with morbidity, disability and mortality, with faster aging in young adults with childhood adversity and effect sizes similar to GrimAge clock. Notably, there is a striking lack of overlap of CpGs sites used in each of these epigenetic aging measures, suggesting they describe different aspects of the biological aging processes (cognitive and functional decline, increased inflammation, etc.) [5].

EAA has been associated with low socioeconomic position (SEP) [10–15] measured by proxy variables including education, area deprivation index, or own and parental occupation. While the association between SEP and epigenetic age acceleration is partially mediated by smoking, alcohol consumption, obesity and other lifestyle-related risk factors for non-communicable diseases, differences in these health behaviours alone do not entirely explain the SEP gradient in epigenetic ageing in adults. Working conditions, including work related stress, are potential contributors to this gradient since work characteristics and known job-stressor indicators are associated with clinical biomarkers and adverse health outcomes. Those who reported higher job strain showed an increased risk of coronary heart disease [16, 17] and diabetes [18], increased brain-derived neurotrophic factor [19] and atherosclerosis in its early non-symptomatic stages [20]. A 2017 review [21] found that effort-reward imbalance at work was associated with biological changes, which are on pathways leading to stress-related conditions including increased heart rate variability and blood pressure, altered blood lipids, immune function and inflammation, and increased cortisol release. EAA has been linked to measures of psychosocial stress such as anxiety and post-traumatic stress disorder [7], suggesting stress induced by working conditions may also affect EAA. Overall based on previous research, EAA has been suggested as an intermediate biological mechanism linking environmental exposures (including stress) with poor health outcomes and mortality later in life.

However, the link between work characteristics and epigenetic ageing remains a relatively unexplored area: only Hughes et al. [11] have investigated current employment with the first-generation clocks. Recently, we reported in the Northern Finnish Birth Cohort 1966 (NFBC) study [22] that those in a job strain-active work (high demand and high control) showed slower aging, assessed through the PhenoAge clock, by around 1.5 years, and white-collar workers had a six month younger epigenetic age, assessed by the GrimAgeAA clock when compared to blue collar workers. Also, working for more than 40 hours per week was associated with an increase in epigenetic age of over 1.5 years, assessed by the first-generation clocks.

Understanding the effects of working conditions on biological aging is of particular relevance due to current changes in working conditions due to rise of the gig-economy work, where gig workers present “alternative work arrangements” for pieces of jobs (“gigs”) or more generally short-term contracts, which are mainly agreed upon via digital platforms for different services [23]. These types of jobs are particularly prevalent among the younger working population, where long-term occupational health studies are still unavailable. Therefore, investigating how related work conditions (including temporary contract, self-employment, working hours and job insecurity) affect EAA biomarkers may pave the way for further health assessments in this younger population.

Here we examined the association of four epigenetic age acceleration biomarkers (Horvath, Hannum, PhenoAge, GrimAge) and pace of aging (DunedinPoAm, DunedinPACE), in relation to employment and the following dimensions of work: job type, job stability; job schedule; autonomy and influence at work; occupational physical activity; and feelings regarding the job. The study sample includes a subset of the British Understanding Society dataset [24] from two cross-sectional waves covering the current workforce.

## RESULTS

### Descriptive summary of epigenetic age acceleration and pace of aging

Table 1 contains detailed definitions and interpretations of the two work stress indicators and the other work characteristics examined and Table 2 reports summary statistics for the study population (*n* = 631), composed of 51% women and in an age range from 26 to 72 years. Women presented the lowest mean epigenetic age acceleration: GrimaAge AA for men was 1.5 (standard deviation sd 4.6) and for women −1.3 (sd 4.2), with men also showing a higher pace of aging (1.03, sd 0.08). Overall, 41% subjects were overweight; men and women presented similar educational levels. A higher percentage of current smokers was present among men (20%), while both sexes had high percentages of heavy alcohol consumers (men: 76%, women: 60%). A great majority of subjects was employed (96%), approximately 81% as employees and 15% as self-employed workers, with only 4.5% unemployed. Of those working as employees, 95% held a permanent position. A high proportion of both men and women reported high job satisfaction (81% and 83%, respectively). According to the NS-SEC, more than 40% workers were employed in management and professional jobs, 15% in intermediate occupations, 13% as small employers, 7.4% in lower supervisory and technical positions, and around 20% as skilled or semi-skilled workers, with some differences by sex.

**Table 1:** Work characteristics definition.

**Work dimension group**	**Definitions of included exposures**
**Job type:**	Five category version of the National Statistics Socio-economic Classification (NS-SEC). Job sector: Organisation is private business/limited company.
**Job stability:**	Contract status classed as permanent/temporary. Employment status as employed/self-employed/unemployed. Job security was assessed as the likelihood of losing job during the next 12 months (likely and very likely/unlikely). Salaried or paid by hour. Presence of second job or not.
**Job schedule:**	Times of the day usually worked have been collapsed into: 'during the day', night/evenings and rotating shifts. Working on weekends was categorized into no weekend working, some weekends, most weekends. Hours worked per week were classified in three groups: less than 40 hours, 40 hours, and more than 40 hours.
**Autonomy and influence at work:**	Managerial duties as: not manager/supervisor (1), foreman/supervisor (2), and manager (3). As a potential proxy of job control, five questions investigated how much control a subject has to influence tasks, work pace, work manner, task order, work hours. Each was rated on a four-point scale. We summed over the answers and split them in three levels: low (15, 20) (the reference), moderate (10, 15) and high (4, 10).
**Occupational physical activity (OPA):**	The physicality of the job was reduced into three categories: very active; fairly active; and not very/not at all physically active at work.
**Feelings regarding the job:**	Current job satisfaction was investigated on a 7-point scale: completely dissatisfied, mostly dissatisfied, somewhat dissatisfied, neither satisfied or dissatisfied, mostly satisfied, somewhat satisfied and completely satisfied. We collapsed in three levels: dissatisfied (completely, mostly and somewhat) (1), neither satisfied or dissatisfied (2) and satisfied (completely, mostly, somewhat) (3). Job feeling were derived from six questions in the format of: ‘Thinking of the past few weeks, how much of the time has your job made you feel: tense, uneasy, worried, depressed, gloomy, miserable?’. We computed the sum of the responses (on five-point scale where 1 means ‘Never’ and 5 means ‘All the time’) and split feelings of job in three levels: positive (6–8) (as reference), average (8–11) and negative (11–30). The categorical class were defined on the quantile cut-offs as the distribution of the total was highly skewed.

**Table 2:** Descriptive statistics of the study population, mean and standard deviation (sd) for continuous variables and frequency and percentage for categorical variables.

	**Levels**	**All *n* = 631**	**Female, *N* = 342**	**Male, *N* = 289**
Epigenetic AA mean (sd)	HorvathAA	0.0 (4.6)	−0.5 (4.4)	0.6 (4.8)
	HannumAA	0.0 (3.5)	−0.8 (3.2)	0.9 (3.7)
	PhenoAA	0.0 (5.7)	0.1 (5.7)	−0.1 (5.8)
	GrimAgeAA	0.0 (4.6)	−1.3 (4.2)	1.5 (4.6)
Pace of aging	DunedinPoAm	1.02 (0.08)	1.01 (0.07)	1.03 (0.08)
	DunedinPACE	1.03(0.13)	1.03(0.13)	1.03(0.14)
Age	(26, 30)	30 (4.8%)	15 (4.4%)	15 (5.2%)
	(30, 40)	133 (21%)	84 (25%)	49 (17%)
	(40, 50)	230 (37%)	124 (36%)	106 (37%)
	(50, 60)	159 (25%)	82 (24%)	77 (27%)
	>60	75 (12%)	35 (10%)	40 (14%)
	Missing	4	2	2
BMI *n* (%)	Optimal <24.9	168 (27%)	105 (32%)	63 (22%)
	Overweight 25–29.9	261 (42%)	127 (38%)	134 (47%)
	Obese >30	188 (30%)	101 (30%)	87 (31%)
	Missing	14	9	5
Education, *n* (%)	Primary (no qualification)	38 (6.0%)	17 (5.0%)	21 (7.3%)
	Secondary (A-level, GCSE, other qualifications)	368 (59%)	202 (59%)	166 (58%)
	Tertiary (degree or other higher degree)	223 (35%)	122 (36%)	101 (35%)
	Missing	2	1	1
Alcohol consumption, *n* (%)	Rarely	64 (11%)	46 (14%)	18 (6.8%)
	Moderate	129 (22%)	82 (25%)	47 (18%)
	Heavy	395 (67%)	194 (60%)	201 (76%)
	Missing	43	20	23
Smoking, *n* (%)	Never	295 (47%)	174 (51%)	121 (42%)
	Past	218 (35%)	110 (32%)	108 (38%)
	Current	115 (18%)	58 (17%)	57 (20%)
	Missing	3	0	3
**Current job classification:**				
NS-SEC	Semi-routine, routine worked/LT unemployed	132 (22%)	83 (26%)	49 (18%)
	Lower supervisory and technical	44 (7.4%)	13 (4.1%)	31 (11%)
	Small employers and own account	79 (13%)	31 (9.7%)	48 (17%)
	Intermediate	88 (15%)	63 (20%)	25 (9.1%)
	Management and professional	252 (42%)	130 (41%)	122 (44%)
	Missing	36	22	14
Job sector	other type of organization	224 (45%)	157 (55%)	67 (31%)
	private firm or business, a limited company	276 (55%)	127 (45%)	149 (69%)
	Missing	131	58	73
**Job stability:**				
Status contract	A permanent job	571 (96%)	307 (95%)	264 (96%)
	not permanent job	26 (4.4%)	15 (4.7%)	11 (4.0%)
	Missing	34	20	14
Status job	paid employment(ft/pt)	508 (81%)	288 (84%)	220 (76%)
	self employed	94 (15%)	39 (11%)	55 (19%)
	unemployed	29 (4.6%)	15 (4.4%)	14 (4.8%)
Job security	Unlikely to lose their job	450 (91%)	256 (91%)	194 (90%)
	Likely to lose their job	45 (9.1%)	24 (8.6%)	21 (9.8%)
	Missing	136	62	74
Pay type	salaried	332 (67%)	179 (63%)	153 (71%)
	paid by the hour	166 (33%)	105 (37%)	61 (29%)
	Missing	133	58	75
Has second job	No			
	Yes	53 (8.4%)	28 (8.2%)	25 (8.7%)
	Missing	3	0	3
**Job Schedule:**				
Working time	During the day	434 (73%)	231 (72%)	203 (75%)
	Night	21 (3.5%)	11 (3.4%)	10 (3.7%)
	Rotating shifts	139 (23%)	80 (25%)	59 (22%)
	Missing	37	20	17
Working weekends	no weekend working	259 (44%)	159 (49%)	100 (37%)
	yes - some weekends	219 (37%)	109 (34%)	110 (40%)
	yes - most/every weekend	116 (20%)	54 (17%)	62 (23%)
	Missing	37	20	17
Working hours	Less than 40 hours	395 (79%)	257 (91%)	138 (64%)
	40 hours	64 (13%)	18 (6.4%)	46 (21%)
	More than 40 hours	41 (8.2%)	8 (2.8%)	33 (15%)
	Missing	131	59	72
**Autonomy and influence at work:**				
Managerial duties	not manager/supervisor	309 (62%)	196 (69%)	113 (52%)
	foreman/supervisor	73 (15%)	40 (14%)	33 (15%)
	manager	119 (24%)	48 (17%)	71 (33%)
	Missing	130	58	72
Job autonomy	(15, 20) High	55 (9.3%)	33 (10%)	22 (8.1%)
	(10, 15) Moderate	149 (25%)	101 (31%)	48 (18%)
	(0, 10) Low	390 (66%)	188 (58%)	202 (74%)
	Missing	37	20	17
**Occupational physical activity:**				
OPA	Not very active/Not at all	239 (40%)	123 (38%)	116 (43%)
	Fairly Active	248 (42%)	147 (46%)	101 (37%)
	Very Active	107 (18%)	52 (16%)	55 (20%)
	Missing	37	20	17
**Feelings regarding the job:**				
Job satisfaction	Neither Satisfied/Dissatisfied	37 (6.2%)	21 (6.5%)	16 (5.9%)
	Dissatisfied	70 (12%)	33 (10%)	37 (14%)
	Satisfied	487 (82%)	268 (83%)	219 (81%)
	Missing	37	20	17
Job feelings	(6, 8) Positive	234 (39%)	119 (37%)	115 (42%)
	(8, 11) Average	165 (28%)	84 (26%)	81 (30%)
	(11, 30) Negative	194 (33%)	118 (37%)	76 (28%)
	Missing	38	21	17

Pearson correlation coefficients among EAA were within the range of 0.1–0.5. The highest was between HannumAA and HorvathAA (r = 0.5), the lowest was between GrimAgeAA and HorvathAA (r = 0.1) (Supplementary Figure 1), while it was higher between the pace of aging markers (r = 0.63). When we investigated dependences among lifestyle variables and job characteristics, education and sex were primarily associated with job sector, occupational class, autonomy, and managerial duties. Smoking was associated with job sector, employment status, and occupational class, while alcohol consumption was associated only with managerial duties.

Smoking and obesity were significantly associated with GrimAgeAA, PhenoAA and both pace of aging measures (Supplementary Figure 2).

### Association between work characteristics and EAA biomarkers

**Figure 1:**
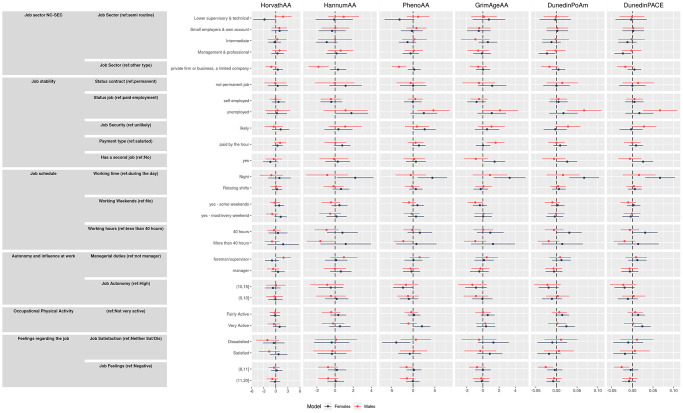
Effect size and 95% confidence intervals for unadjusted and adjusted model (interpretable as years of increase/decreasing epigenetic age) of the association between primary outcomes and the four epigenetic aging biomarkers and the pace of aging measures.

Results from the unadjusted and adjusted models (adjustment was made for sex, alcohol consumption, smoking, BMI, and educational level) for all the epigenetic aging measures are shown in Figure 1 and in Supplementary Table 1 and Table 3, respectively. Below we describe results from the fully adjusted analyses for each work dimension.

**Table 3:** Adjusted linear regression estimated coefficient with 95% confidence intervals for the epigenetic age acceleration and pace of aging.

	**Levels**	**HorvathAA**	**HannumAA**	**PhenoAA**	**GrimaAgeAA**	**DunedinPoAm**	**DunedinPACE**
**Current job classification:**							
NS-SEC	Semi-routine, routine worked/LT unemployed	Ref.	Ref.	Ref.	Ref.	Ref.	Ref.
	Lower supervisory and technical	0.158 (−1.426, 1.743)	0.491 (−0.724, 1.706)	−0.552 (−2.504, 1.399)	0.475 (−0.696, 1.646)	−0.004 (−0.026, 0.017)	0 (−0.044, 0.045)
	Small employers and own account	0.588 (−0.734, 1.91)	−0.302 (−1.316, 0.712)	−0.083 (−1.711, 1.546)	−0.261 (−1.238, 0.716)	0 (−0.018, 0.018)	−0.037 (−0.074, 0.001)
	Intermediate	0.059 (−1.204, 1.321)	−0.511 (−1.48, 0.457)	−0.758 (−2.313, 0.797)	−0.043 (−0.977, 0.89)	−0.009 (−0.026, 0.009)	−0.016 (−0.051, 0.019)
	Management and professional	0.517 (−0.582, 1.616)	0.289 (−0.554, 1.132)	−0.496 (−1.85, 0.858)	−0.25 (−1.063, 0.562)	−0.013 (−0.028, 0.002)	−0.046 (−0.077, −0.016)^*^
Job sector	other type of organization	Ref.	Ref.	Ref.	Ref.	Ref.	Ref.
	private firm or business, a limited company	−0.08 (−0.965, 0.805)	−0.562 (−1.235, 0.112)	−1.072 (−2.152, 0.007)	−0.279 (−0.921, 0.362)	−0.004 (−0.016, 0.007)	−0.017 (−0.042, 0.008)
**Job stability:**							
Status contract	A permanent job	Ref.	Ref.	Ref.	Ref.	Ref.	Ref.
	not permanent job	0.042 (−1.722, 1.806)	0.56 (−0.791, 1.911)	−0.307 (−2.47, 1.856)	0.583 (−0.718, 1.885)	0.006 (−0.018, 0.03)	0.012 (−0.038, 0.061)
Status job	paid employment(ft/pt)	Ref.	Ref.	Ref.	Ref.	Ref.	Ref.
	self employed	0.129 (−0.897, 1.154)	−0.511 (−1.293, 0.27)	0.031 (−1.231, 1.293)	−0.538 (−1.282, 0.207)	0.004 (−0.01, 0.018)	−0.013 (−0.041, 0.016)
	unemployed	0.316 (−1.571, 2.204)	1.518 (0.08, 2.956)^*^	3.212 (0.89, 5.533)^*^	1.535 (0.165, 2.905)^*^	0.036 (0.01, 0.062)^*^	0.045 (−0.008, 0.097)
Job security	Unlikely to lose their job	Ref.	Ref.	Ref.	Ref.	Ref.	Ref.
	Likely to lose their job	0.295 (−1.204, 1.794)	0.698 (−0.451, 1.847)	1.826 (−0.007, 3.659)	0.829 (−0.256, 1.915)	0.01 (−0.01, 0.03)	0.028 (−0.014, 0.07)
Pay type	salaried	Ref.	Ref.	Ref.	Ref.	Ref.	Ref.
	paid by the hour	0.648 (−0.274, 1.571)	0.439 (−0.264, 1.142)	0.927 (−0.203, 2.057)	0.611 (−0.058, 1.279)	0.005 (−0.007, 0.018)	0.018 (−0.008, 0.043)
Has second job	No	Ref.	Ref.	Ref.	Ref.	Ref.	Ref.
	Yes	−0.761 (−2.051, 0.53)	0.18 (−0.809, 1.17)	0.18 (−1.419, 1.78)	0.335 (−0.608, 1.279)	0.012 (−0.005, 0.03)	0.006 (−0.03, 0.042)
**Job Schedule:**							
Working time	During the day	Ref.	Ref.	Ref.	Ref.	Ref.	Ref.
	Night	0.042 (−1.913, 1.997)	0.735 (−0.762, 2.232)	2.009 (−0.382, 4.401)	2.117 (0.685, 3.549)^*^	0.044 (0.017, 0.07)^*^	0.061 (0.006, 0.115)^*^
	Rotating shifts	0.18 (−0.712, 1.072)	0.241 (−0.442, 0.924)	0.144 (−0.948, 1.235)	−0.06 (−0.714, 0.593)	0.005 (−0.007, 0.017)	0.001 (−0.024, 0.026)
Working weekends	no weekend working	Ref.	Ref.	Ref.	Ref.	Ref.	Ref.
	yes − some weekends	0.408 (−0.429, 1.244)	0.067 (−0.574, 0.709)	0.163 (−0.863, 1.19)	−0.647 (−1.262, −0.032)	−0.003 (−0.014, 0.008)	−0.007 (−0.031, 0.016)
	yes − most/every weekend	0.208 (−0.814, 1.23)	−0.164 (−0.948, 0.619)	0.285 (−0.968, 1.539)	−0.055 (−0.807, 0.697)	−0.003 (−0.016, 0.011)	−0.007 (−0.036, 0.022)
Working hours	Less than 40 hours	Ref.	Ref.	Ref.	Ref.	Ref.	Ref.
	40 hours	−0.158 (−1.468, 1.153)	−0.396 (−1.393, 0.601)	0.289 (−1.312, 1.891)	0.429 (−0.52, 1.379)	0.006 (−0.011, 0.024)	−0.033 (−0.07, 0.003)
	More than 40 hours	−0.446 (−2.057, 1.166)	−1.158 (−2.383, 0.068)	−1.496 (−3.465, 0.473)	−0.471 (−1.638, 0.697)	−0.012 (−0.034, 0.009)	−0.045 (−0.09, 0)
**Autonomy and influence at work:**							
Managerial duties	not manager/supervisor	Ref.	Ref.	Ref.	Ref.	Ref.	Ref.
	foreman/supervisor	0.245 (−0.959, 1.448)	0.414 (−0.504, 1.332)	0.521 (−0.952, 1.994)	0.318 (−0.553, 1.19)	0.01 (−0.006, 0.026)	0.003 (−0.031, 0.037)
	manager	−0.141 (−1.208, 0.927)	0.245 (−0.569, 1.059)	−0.359 (−1.665, 0.948)	−0.385 (−1.158, 0.388)	−0.007 (−0.021, 0.007)	−0.021 (−0.051, 0.009)
Job autonomy	(15, 20) High	Ref.	Ref.	Ref.	Ref.	Ref.	Ref.
	(10, 15) Medium	−0.579 (−1.99, 0.832)	−0.743 (−1.821, 0.336)	−2.224 (−3.943, −0.505)	−1.001 (−2.039, 0.037)	−0.02 (−0.039, −0.001)^*^	−0.079 (−0.118, −0.04)^*^
	(0, 10) Low	−0.276 (−1.563, 1.012)	−0.14 (−1.124, 0.845)	−1.061 (−2.63, 0.508)	−0.395 (−1.342, 0.552)	−0.006 (−0.023, 0.011)	−0.045 (−0.081, −0.01)^*^
**Occupational physical activity**							
OPA	Not very active/Not at all	Ref.	Ref.	Ref.	Ref.	Ref.	Ref.
	Fairly Active	−0.238 (−1.072, 0.595)	−0.022 (−0.661, 0.618)	−0.145 (−1.168, 0.878)	0.366 (−0.248, 0.981)	0.011 (−0.001, 0.022)	0.018 (−0.006, 0.041)
	Very Active	0.14 (−0.927, 1.206)	0.081 (−0.737, 0.898)	0.309 (−0.999, 1.617)	0.549 (−0.237, 1.336)	0.014 (0, 0.029)^*^	0.032 (0.002, 0.061)^*^
**Feelings regarding the job**							
Job satisfaction	Neither Satisfied/Dissatisfied	Ref.	Ref.	Ref.	Ref.	Ref.	Ref.
	Dissatisfied	−1.037 (−2.913, 0.839)	−0.198 (−1.639, 1.243)	−1.551 (−3.851, 0.749)	0.603 (−0.785, 1.99)	−0.001 (−0.027, 0.024)	−0.022 (−0.075, 0.03)
	Satisfied	−0.061 (−1.671, 1.549)	−0.368 (−1.605, 0.869)	−0.294 (−2.268, 1.679)	0.421 (−0.77, 1.612)	−0.009 (−0.031, 0.013)	−0.029 (−0.074, 0.017)
Job feelings	(6, 8) Positive	Ref.	Ref.	Ref.	Ref.	Ref.	Ref.
	(8, 11) Average	−0.125 (−1.041, 0.791)	−0.319 (−1.022, 0.383)	−0.706 (−1.829, 0.417)	−0.463 (−1.14, 0.213)	−0.016 (−0.028, −0.004)	−0.029 (−0.054, −0.003)^*^
	(11, 30) Negative	−0.564 (−1.447, 0.319)	−0.349 (−1.026, 0.328)	−0.641 (−1.723, 0.442)	−0.241 (−0.893, 0.411)	−0.009 (−0.021, 0.003)	−0.003 (−0.028, 0.021)

Fully adjusted models for: BMI, alcohol consumption, educational level, sex and smoking. ^*^p value < 0.05.

#### Job type

Jobs classed as management or professional by NC-SEC showed negative directions of associations with the second-generation clocks and DunedinPoAm compared to routine worker positions although all confidence intervals overlapped zero. However, significant results were found for DunedinPACE, with a deceleration of 4.6% (95% CI −0.077, −0.016). This is in contrast to unadjusted results where a clear trend across categories was observed for these clocks, suggesting a strong influence of education (a proxy for early and mid-life SEP) on the effects of job classification. Estimates for working in a private firm showed negative associations with all clocks, although all confidence intervals overlapped zero.

#### Job stability

Being unemployed showed the strongest effects of all exposure tested: positive directions of effect were observed across all clocks among the unemployed compared to those in paid employment, with HannumAA increased by 1.51 years (95% CI 0.088, 2.95), PhenoAA by 3.21 years (95% CI 0.89, 5.53), GrimaAgeAA by 1.53 years (95% CI 0.16, 2.90) and DunedinPoAm by 0.036 (95% CI 0.01, 0.06). No consistent effects were observed for being self-employed.

Workers who felt job insecurity, i.e., a threat of losing their job in the next year were likely to show a positive direction of associations across all clocks, with PhenoAA increased by 1.83 years (95% CI −0.007, 3.66) compared to those in secure positions. Working in a temporary job showed little association across all clocks. Being paid by the hour showed a positive direction of associations across all clocks while having a second job showed a positive direction of associations across all clocks except HorvathAA.

#### Job schedule

Night working showed a positive direction of associations across all clocks, with increases in GrimaAgeAA of 2.12 years (95% CI 0.68, 3.55) and DunedinPoAm by 0.04 (95% CI 0.02, 0.08) and by 0.06 (95% CI 0.006, 0.115) for DunedinPACE. Inconsistent results were observed for rotating shifts and weekend working. Interestingly, negative directions of associations were observed for all clocks with working more than 40 hours/week, although the effect was not statistically significant.

#### Autonomy and influence at work

Compared to not having supervision or management responsibilities, a positive direction of association was observed for all clocks for those working in a supervisory role, and negative directions were observed for all clocks except HannumAA for those in a management role, although all confidence intervals overlapped zero. Perhaps unexpectedly, compared to having high job autonomy, medium and low autonomy jobs were associated with negative associations across all clocks. The effect of medium autonomy was for PhenoAA −2.22 years (95% CI −3.94, −0.50), for DunedinPoAm −0.02 years (95% CI −0.039, −0.001) and for DunedinPACE −0.079 years (95% CI 0.118, 0.04).

#### Occupational physical activity

Being physically very active at work was associated with age acceleration across all clocks although the associations were not statistically significant, except in the adjusted model for DunedinPoAm, where it was associated with an increase by 0.014 years (95% CI 0.0001, 0.029), and for DunedinPACE, showing an increase of 0.032 years (95% CI 0.002, 0.061).

#### Feelings regarding the job

Generally, inconsistent results were observed for job satisfaction. Regarding overall feeling towards their job, unexpectedly, negative directions of effect were observed for all clocks for those who had average or negative feelings, compared to those with a positive feeling. For those who had average feelings towards their job, DunedinPACE was decreased by 0.02 (95% CI −0.054, −0.003).

### Sensitivity analysis: sex-specific adjusted models

**Figure 2:**
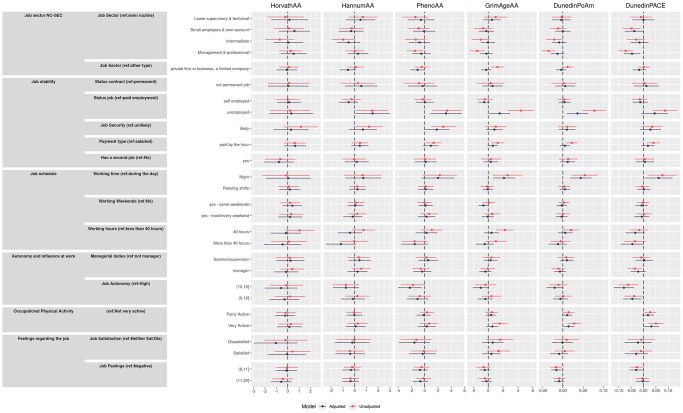
Effect size and 95% confidence intervals for adjusted males and females models (interpretable as years of increase/decreasing epigenetic age) of the association between primary outcomes and the four epigenetic aging biomarkers and the pace of aging.

In stratified analysis, there were some notable differences in the sex-specific adjusted models (Figure 2 and Supplementary Tables 2, 3). Among women, stronger effects than for men were observed with the DunedinPoAm (−0.023 95% CI −0.042, −0.003) for women working in managerial positions compared to semi-routine positions. Also, among women only, deceleration was observed for those in lower supervisory roles for HorvathAA (−2.78 95% CI −5.50, −0.05). Working in a private firm appeared more beneficial for men with an estimate that was associated with a slower aging by almost two years for HannumAA (−1.82 95% CI −2.90, −0.74) and by over three years for PhenoAA (−3.25 95% CI −4.86, −1.63). Being unemployed was associated with greater age acceleration for all measures except HannumAA among men, with the strongest effects on men noted for PhenoAA (4.7 years, 95% CI 1.03, 8.35) and DunedinPoAm (0.067 95% CI 0.026, 0.108).

Longer working hours were generally associated with slower ageing in men and faster ageing in women: specifically in men working more than 40 hours per week slowed substantially the biological age for PhenoAA (−2.23 95% CI −4.43, −0.04) and HannumAA(−1.62 95% CI −3.06, −0.17), while also the other biomarkers showed negative coefficient signs. Effects of night working on accelerated aging appeared to be driven by effects in women, with effects observed in women for HannumAA (2.23 years 95% CI 0.21, 4.22), PhenoAA (4.45 years 95% CI 1.11, 7.90), GrimAgeAA (3.30 95% CI 1.37, 5.23), DunedinPoAm (0.067 95% CI 0.032, 0.103) and DunedinPACE (0.061 95% CI 0.006, 0.115).

Finally, being very physically active at work appeared to increase epigenetic aging only for women, by 2.04 years (95% CI 0.15, 3.93) for PhenoAA and by 0.024 years (95% CI 0.003, 0.044) for DunedinPoAm and for DunedinPACE increases of 0.03 (95% CI 0.001, 0.058) and 0.056 (95% CI 0.017, 0.095) for fairly and very active categories respectively, were observed among women.

## DISCUSSION

We have assessed the magnitude and association of accelerated epigenetic aging and pace of ageing with characteristics of employment and work. For the job characteristics studied, the range observed was within ± 1.5 years, with highest values of accelerated epigenetic aging for unemployed subjects, particularly by using GrimaAge (up to 4.3 years) and PhenoAge (3.3 years), even after adjusting for known risk factors. In addition, low job security accelerated aging by 1.8 years with PhenoAA, and night working increased aging up to 2.1 years with GrimAge and increased the rate of aging by 4% according to DunedinPoAm and 6% for DunedinPACE. Furthermore, differences among associations between sexes were observed particularly for unemployment, working hours, night working and physical activity at work.

Our results differ from the analysis conducted by Hughes et al. [11] who also used the Understanding society study. In their paper, they found that unemployment and duration of unemployment over the past 12 years was not associated with HannumAA and HorvathAA. In our analysis, we found increases in HannumAA among the unemployed and stronger effects with the second-generation clocks. This difference may be due to the exclusion of retired, sick, and disabled subjects, and family carers and comparison to those in paid employment only in our study. However, the unemployed subset includes 4.5% of the sample and the small size data prevents generalization of these results to the population level, and further studies will be needed to assess reproducibility of the observed effects. Chandola et al. [25] using the larger subset of the UKLHS cross-sectional waves (*n* = 6, 025) reported that the unemployed who found a job perceived as of poor quality showed increased inflammatory biomarkers, compared to the group that was still unemployed. Ala-Mursula et al. found that long-term unemployment in early adulthood is associated with shorter telomere length among men, but not women in the NFBC study [26]. Similarly, we observed stronger effects of unemployment on aging biomarkers among men.

We observed that perceived job insecurity, i.e., fear of unemployment, was also associated with accelerated epigenetic age by almost two years as measured with the PhenoAge clock. A larger study [27] with the Understanding Society dataset found that economic insecurity was associated with adverse metabolic and inflammatory biomarkers (particularly HDL-cholesterol, triglycerides, and C- reactive protein), heightening risk for a range of health conditions. A study [28] of civil servant workers in UK reported an association between job insecurity and risk of coronary heart disease, with factors such as health behaviours and sleep disturbances not found to play a role as potential mediator factors. The authors hypothesized that the association could be partially explained by psychological distress, and EAA has been found to be sensitive to mood disorders in previous studies, including depression [29], anxiety and lack of psychosocial support [30].

Our observation on the effects of night shift working confirm the findings of White et al. [16] in a sample of non-Hispanic White women aged 35–74, where working overnight for >10 years corresponded to a PhenoAA of around three years. We were not able to assess for how long the night shift work took place, but it seems that there could be a cumulative effect increasing AA over time for PhenoAge and GrimAge biomarkers. Night shift work is hypothesized to induce circadian disruption and sleep/fatigue interruption and is associated with risk of age-related diseases [31, 32] and breast cancer [33]. Effects have also been reported on specific epigenetic markers in genes involved in circadian regulation [34]. Our study further suggests that effects on epigenetic ageing appear stronger in women, possibly related to psychological effects of non-regular working hours in women [18] or potential sex-specific biological mechanisms [35].

For men, HannumAA was associated with a slowing effect for working more than 40 hours (−1.5 years), in contrast to directions of effect in women. In a similar study [22] that we conducted in the NFBC study at the age of 46 years, we found among women that working more than 40 hours per week was associated with an accelerated aging in both HannumAA and HorvathAA whereas no significant results were observed in relation to working hours for men. A previous study conducted in the Understanding Society study found among women working long hours and weekends apparently deteriorated mental health and increased depressive symptoms [18]. Women typically work longer hours in unpaid domestic work and therefore excess hours in paid work may lead to very long total working hours [19]. A WHO report [36] suggested the pathway between exposure to long working hours (≥55 hours/week) and adverse health outcomes involved physiological and behavioural responses as mediators.

Previous studies have found that there are significant differences between women and men for epigenetic markers. Singmann et al. [20] have found that CpG’s are differentially methylated between men and women, and this may influence how clocks are constructed. This was also confirmed in a study conducted in the USA by Tajuddin et al. [37]. They compared EAA by sex, race (white and African American), poverty level and other environmental factors, concluding that men have a higher age acceleration than women and that African Americans have more widespread methylation changes than whites. Race and sex interact with underlying biological age acceleration, suggesting that altered DNA methylation patterns may be important in age-associated health disparities.

There are several limitations to this work. The cross-sectional UKLHS data selection on the participants excluded other ethnicities; therefore, a possible bias could be introduced for the generalization of these results to the reference population, also considering the small sample size. Furthermore, the study population was rather homogenous with permanent contracts and highly satisfying jobs, that limited any attempt at clustering individuals based on job characteristics, as it led to highly unbalanced groups. We were unable to evaluate workers with informal working arrangements, which is of particular relevance to the gig-economy [23, 38]. We lacked accurate job descriptions such as job titles or other information that could help define specific workers’ profiles. The Understanding Society waves used in this study comprised information from wave 2010–2011 integrated with waves 2011–12 and 2012–13; therefore, we could not establish a perfect correspondence between the health assessment and some job indicators, as some time lag may have occurred. Additionally, the panel survey structure prevents linking potential health outcomes to the health measurements already recorded. While the Understanding Society panel collects a rich amount of data, the survey does not include known job stressor scales. The items investigating feelings on the job and job autonomy were used as proxies of exposure to work stressors. However, the interpretation remains incomplete as known psychosocial measurement scales such as job strain [38] or effort-reward imbalance [39] are composed of other dimensions such as job demand, control, reward, and overcommitment. Lastly, we could not account for leisure-time physical activity and income, as they were unavailable for data subsets with DNA methylation.

In conclusion, our study suggests that being unemployed, in insecure employment, or working night shifts may increase age acceleration and the rate of aging, suggesting the need for policy interventions for tackling these social inequalities in health and ageing. Potentially different patterns of association and biomarker levels were found for men and women regarding job characteristics and epigenetic acceleration age. Given the recent rapid changes in job arrangements, job places and work-life balance, we believe that further studies should be carried out to evaluate those in more vulnerable working situations, aiming to provide further evidence to improve work policies.

## METHODS

### Study population

The study sample consisted of participants from the United Kingdom Household Panel Study (UKHLS), also known as Understanding Society [40]; an ongoing longitudinal, nationally representative study of the UK, designed as a two-stage stratified random sample of the general population. While Understanding Society is a panel survey, the data used here consist of two pooled cross-sectional waves where a nurse collected blood samples from the respondents, among other physiological measures. The eligibility criteria for collecting blood samples were: (i) participation in the previous main interviews in England (had participated in all annual interviews between 1999 (BHPS wave 9) and 2011–2013 (Understanding Society wave 2 and 3); (ii) age 16 and over; (iii) living in England, Wales, or Scotland. From the potential pool of 6337 survey respondents, eligibility requirements for epigenetic analyses meant that the samples for DNA methylation measurement were restricted to participants of white ethnicity, resulting in 1175 subjects [41]. We have included only those in the current labour force, excluding retired, sick, and disabled subjects, and family carers. We were left with *n* = 631 subjects who were employees (part or full time), self-employed, or unemployed.

### DNA collection

Methylation profiling has been conducted on DNA samples using the Illumina Methylation EPIC BeadChip which integrates over 850,000 Methylation sites across the genome. A five hundred–nanogram samples of whole-blood DNA from 1,175 persons were treated with sodium bisulfite using the EZ96 DNA methylation kit (Zymo Research, Irvine, CA, USA) following the manufacturer’s standard protocol. DNA methylation was assessed using the Illumina Infinium HumanMethylationEPIC BeadChip kit (Illumina, Inc., San Diego, CA, USA) [42]. Raw signal intensities were pre-processed, normalised, and converted into β values using the Bioconductor bigmelon package [43].

### Computation of epigenetic clock measures

We have calculated four epigenetic age indicators: Horvath DNAm age [1] based on a weighted average of 353 age-related CpGs; Hannum DNAm age [2] based on 71 blood specific age-related CpGs; PhenoAge DNAm age [44] is based on 513 phenotypic age-related CpGs and DNAm GrimAge [4] was derived in a multi-step process from 1,030 CpGs. We computed the extrinsic epigenetic age acceleration (EAA) as the residual values of the linear regression of epigenetic age on chronological age (HorvathAA, HannumAA, PhenoAA, and GrimAgeAA). In addition to the EAA measures, which assess biological age at a single time point, we have investigated the rate of increasing biological age through the DunedinPoAm [8], DunedinPACE based on 46 and 173 CpGs, respectively.

### Work-related indicators

We used all questionnaire items that could indicate job characteristics, within the categories of job type (e.g., National Statistics Socio-economic Classification (NS-SEC) and job sector), job stability (e.g., type of contract, job security); job schedule (e.g., hours worked); autonomy and influence at work (e.g. job control, managerial duties); occupational physical activity; and feelings regarding the job (e.g., job satisfaction).

### Covariates

As additional variables, we have included lifestyle-related risk factors. Smoking was classified as: never, past, and current smoker. Alcohol consumption was categorised based on frequency of alcoholic drink consumption during the last 12 months as rarely (once or twice a year, not at all), moderate (once or twice a month, once every couple of months) and heavy (almost every day, five or six days a week, three or four days a week). Body Mass Index (BMI) is presented in three levels (optimal <24.9, overweight 25–29.9, obese ≥30.0); educational level was classified as primary (no qualification), secondary (A-level, GCSE, other qualifications) or tertiary (university degree or other higher degree).

### Statistical methods

We computed descriptive statistics (mean and standard deviation) for all the continuous variables and proportions for categorical variables. We computed Pearson correlation coefficients for the four EAA measures and DunedinPoAm and DunedinPACE.

For each job characteristics, we fitted linear regression models to evaluate the association of the epigenetic clocks and pace of aging with known health risk factors and work characteristics. We estimated unadjusted and fully adjusted models (adjustment was made for sex, alcohol consumption, smoking, BMI and educational level). Age was not adjusted for because chronological age has zero correlation with age acceleration measures (by definition). Results are reported as point estimates and 95% confidence intervals. The estimates are interpretable as years of increase/decrease for epigenetic age; positive coefficients indicate age acceleration, and negative coefficients indicate a decrease of the estimated biological age compared to the chronological age. For the pace of ageing marker, the rate indicates the percentage of biological aging increase compared to the reference category. We expect that job indicators on the well-being side should have negative or at least null estimated coefficients, while indicators of adverse working conditions would present positive estimates. We estimated sex-specific fully adjusted linear regression models as a sensitivity analysis. We assessed linear regression assumptions on residual analysis. The clocks have different standard deviations, therefore is not possible to compare the results by standardizing the estimated effects. Given the descriptive and exploratory approach, we abstained from *p*-value adjustments to correct for multiple testing.

## Supplementary Materials

Supplementary Figures

Supplementary Tables
