# Supplementary material for: Association between work characteristics and epigenetic age acceleration: cross-sectional results from UK – Understanding Society study
Source: Aging (Albany NY). 2022 Oct 5;14(19):7752–73. doi: 10.18632/aging.204327 (PMC9596217; doi:10.18632/aging.204327)
Supplement: Supplementary Figures [file aging-14-204327-s001.pdf]

SUPPLEMENTARY FIGURES

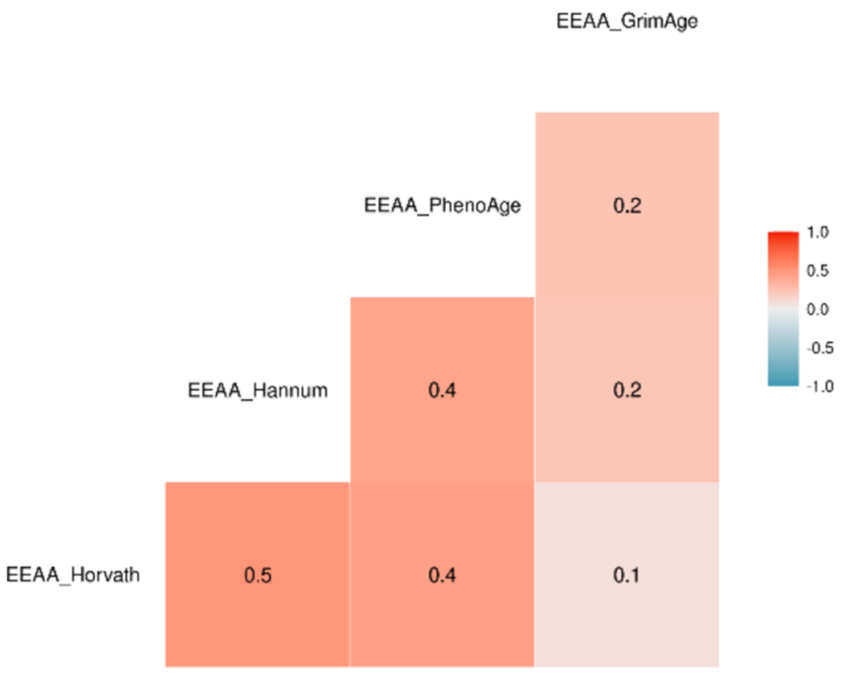

Supplementary Figure 1. Epigenetic age acceleration correlation heatmap, with correlation values.

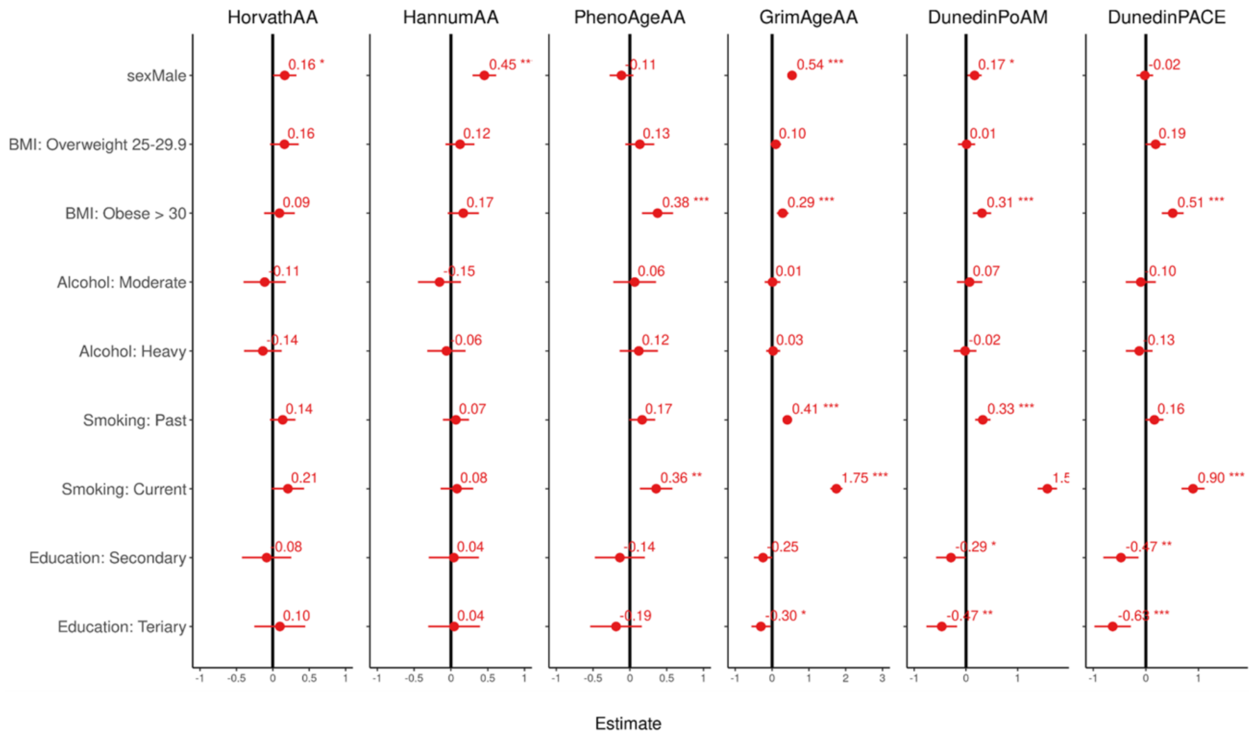

Supplementary Figure 2. Effect size and 95% confidence intervals (interpretable as years of increase/decreasing epigenetic age) of the regression coefficients between the four epigenetic aging biomarkers and the pace of aging markers and risk factors.
