# Supplementary material for: Association between work characteristics and epigenetic age acceleration: cross-sectional results from UK – Understanding Society study
Source: Aging (Albany NY). 2022 Oct 5;14(19):7752–73. doi: 10.18632/aging.204327 (PMC9596217; doi:10.18632/aging.204327)
Supplement: Supplementary Tables [file aging-14-204327-s002.pdf]

SUPPLEMENTARY TABLES

Supplementary Table 1. Unadjusted linear regression estimated coefficients with 95% confidence intervals for the epigenetic age and pace of aging.

	Levels	HorvathAA	HannumAA	PhenoAA	GrimaAgeAA	DunedinPoAm	DunedinPACE
**Current job classification:**							
NS-SEC	Semi-routine, routine worked/LT unemployed	Ref.	Ref.	Ref.	Ref.	Ref.	Ref.
	Lower supervisory and technical	–0.196 (–1.782, 1.39)	0.7 (–0.509, 1.909)	–1.384 (–3.338, 0.571)	1.003 (–0.517, 2.523)	–0.003 (–0.028, 0.021)	–0.01 (–0.053, 0.034)
	Small employers and own account	0.101 (–1.195, 1.397)	–0.246 (–1.234, 0.742)	–0.94 (–2.538, 0.657)	–0.709 (–1.951, 0.533)	–0.012 (–0.032, 0.007)	–0.048 (–0.083, –0.012)*
	Intermediate	–0.695 (–1.949, 0.558)	–0.965 (–1.921, –0.009)*	–1.336 (–2.881, 0.21)	–1.015 (–2.217, 0.187)	–0.021 (–0.04, –0.002)*	–0.03 (–0.064, 0.005)
	Management and professional	0.248 (–0.731, 1.226)	0.018 (–0.729, 0.764)	–1.454 (–2.66, –0.247)*	–1.471 (–2.409, –0.532)*	–0.035 (–0.05, –0.02)*	–0.072 (–0.099, –0.045)*
Job sector	Other type of organization	Ref.	Ref.	Ref.	Ref.	Ref.	Ref.
	Private firm or business, a limited company	0.092 (–0.753, 0.937)	0.096 (–0.545, 0.737)	–0.433 (–1.467, 0.601)	1.243 (0.454, 2.032)*	0.012 (–0.001, 0.025)	0.001 (–0.022, 0.025)
**Job stability:**							
Status contract	A permanent job	Ref.	Ref.	Ref.	Ref.	Ref.	Ref.
	Not permanent job	0.119 (–1.713, 1.951)	0.267 (–1.13, 1.664)	–0.825 (–3.078, 1.428)	0.303 (–1.468, 2.073)	0.002 (–0.026, 0.031)	0.004 (–0.047, 0.055)
Status job	Paid employment(ft/pt)	Ref.	Ref.	Ref.	Ref.	Ref.	Ref.
	Self employed	0.005 (–1.02, 1.03)	–0.254 (–1.033, 0.525)	–0.275 (–1.532, 0.981)	–0.442 (–1.44, 0.557)	0.003 (–0.014, 0.019)	–0.015 (–0.044, 0.014)
	Unemployed	0.215 (–1.528, 1.958)	1.477 (0.152, 2.802)*	3.364 (1.227, 5.5)*	4.37 (2.672, 6.067)*	0.077 (0.05, 0.105)*	0.087 (0.038, 0.136)*
Job security	Unlikely to lose their job	Ref.	Ref.	Ref.	Ref.	Ref.	Ref.
	Likely to lose their job	1.158 (–0.308, 2.623)	1.223 (0.111, 2.334)*	2.806 (1.02, 4.591)*	1.005 (–0.375, 2.384)	0.011 (–0.012, 0.033)	0.034 (–0.007, 0.075)
Pay type	Salaried	Ref.	Ref.	Ref.	Ref.	Ref.	Ref.
	Paid by the hour	0.611 (–0.281, 1.504)	0.448 (–0.225, 1.121)	1.331 (0.244, 2.418)*	1.267 (0.432, 2.101)*	0.022 (0.008, 0.036)*	0.04 (0.015, 0.065)*
Has second job	No	Ref.	Ref.	Ref.	Ref.	Ref.	Ref.
	Yes	–0.656 (–1.965, 0.653)	–0.044 (–1.044, 0.956)	0.034 (–1.586, 1.655)	0.348 (–0.955, 1.65)	0.013 (–0.009, 0.034)	0.005 (–0.032, 0.042)
**Job schedule:**							
Working time	During the day	Ref.	Ref.	Ref.	Ref.	Ref.	Ref.
	Night	–0.149 (–2.192, 1.894)	0.667 (–0.891, 2.224)	2.271 (–0.241, 4.782)	2.605 (0.642, 4.568)*	0.054 (0.022, 0.086)*	0.076 (0.019, 0.133)*
	Rotating shifts	0.09 (–0.801, 0.981)	0.214 (–0.465, 0.894)	0.163 (–0.932, 1.258)	–0.154 (–1.011, 0.702)	0.003 (–0.011, 0.017)	–0.005 (–0.029, 0.02)
Working weekends	No weekend working	Ref.	Ref.	Ref.	Ref.	Ref.	Ref.
	Yes - some weekends	0.199 (–0.64, 1.039)	0.118 (–0.523, 0.758)	–0.032 (–1.065, 1.002)	0.087 (–0.724, 0.898)	0.003 (–0.01, 0.016)	–0.002 (–0.025, 0.022)
	Yes - most/ Every weekend	0.307 (–0.714, 1.328)	0.217 (–0.562, 0.997)	0.691 (–0.566, 1.949)	0.515 (–0.472, 1.502)	0 (–0.016, 0.016)	0.002 (–0.027, 0.031)
Working hours	Less than 40 hours	Ref.	Ref.	Ref.	Ref.	Ref.	Ref.
	40 hours	1.037 (–0.226, 2.3)	0.757 (–0.202, 1.716)	1.072 (–0.474, 2.618)	2.218 (1.044, 3.393)*	0.02 (0, 0.039)*	–0.017 (–0.053, 0.018)
	More than 40 hours	0.102 (–1.436, 1.64)	–0.045 (–1.213, 1.122)	–1.507 (–3.389, 0.376)	1.019 (–0.411, 2.45)	–0.005 (–0.029, 0.019)	–0.035 (–0.078, 0.008)

**Autonomy and influence at work:**							
Managerial duties	Not manager/supervisor	Ref.	Ref.	Ref.	Ref.	Ref.	Ref.
	Foreman/supervisor	0.168<br>(–1.054, 1.39)	0.332<br>(–0.594, 1.258)	0.245<br>(–1.253, 1.742)	0.424<br>(–0.728, 1.576)	0.008<br>(–0.011, 0.027)	–0.009<br>(–0.043, 0.025)
	Manager	0.395<br>(–0.618, 1.408)	0.551<br>(–0.216, 1.319)	–0.581<br>(–1.823, 0.66)	–0.076<br>(–1.031, 0.879)	–0.011<br>(–0.026, 0.005)	–0.036<br>(–0.064, –0.008)*
Job autonomy	(15, 20) High	Ref.	Ref.	Ref.	Ref.	Ref.	Ref.
	(10, 15) Medium	–0.125<br>(–1.568, 1.317)	–0.748<br>(–1.841, 0.345)	–1.745<br>(–3.516, 0.025)	–0.372<br>(–1.763, 1.018)	–0.011<br>(–0.033, 0.012)	–0.062<br>(–0.103, –0.022)*
	(0, 10) Low	0.186<br>(–1.131, 1.502)	0.242<br>(–0.755, 1.24)	–0.75<br>(–2.367, 0.866)	0.431<br>(–0.839, 1.7)	0.001<br>(–0.019, 0.022)	–0.038<br>(–0.075, –0.001)*
**Occupational physical activity**							
OPA	Not very active/<br>Not at all	Ref.	Ref.	Ref.	Ref.	Ref.	Ref.
	Fairly Active	–0.159<br>(–0.988, 0.669)	–0.059<br>(–0.691, 0.574)	0.363<br>(–0.658, 1.383)	0.466<br>(–0.33, 1.261)	0.014<br>(0.002, 0.027)*	0.027<br>(0.004, 0.05)*
	Very Active	0.281<br>(–0.782, 1.344)	0.27<br>(–0.541, 1.081)	0.646<br>(–0.663, 1.956)	1.53<br>(0.509, 2.551)*	0.028<br>(0.012, 0.045)*	0.05<br>(0.021, 0.08)*
**Feelings regarding the job**							
Job satisfaction	Neither Satisfied/<br>Dissatisfied	Ref.	Ref.	Ref.	Ref.	Ref.	Ref.
	Dissatisfied	–0.186<br>(–2.043, 1.671)	–0.058<br>(–1.475, 1.359)	–1.256<br>(–3.541, 1.03)	1.55<br>(–0.242, 3.341)	0.009<br>(–0.02, 0.038)	–0.009<br>(–0.061, 0.043)
	Satisfied	0.417<br>(–1.141, 1.975)	–0.41<br>(–1.599, 0.779)	–0.131<br>(–2.049, 1.787)	1.363<br>(–0.14, 2.866)	0.005<br>(–0.02, 0.029)	–0.01<br>(–0.054, 0.033)
Job feelings	[6, 8) Positive	Ref.	Ref.	Ref.	Ref.	Ref.	Ref.
	(8, 11) Average	–0.059<br>(–0.989, 0.87)	–0.207<br>(–0.916, 0.503)	–0.465<br>(–1.611, 0.68)	–0.636<br>(–1.534, 0.262)	–0.017<br>(–0.031, –0.002)*	–0.026<br>(–0.052, 0)
	(11, 30) Negative	–0.423<br>(–1.311, 0.464)	–0.358<br>(–1.036, 0.319)	–0.2<br>(–1.295, 0.894)	–0.428<br>(–1.285, 0.43)	–0.009<br>(–0.023, 0.004)	–0.005<br>(–0.029, 0.02)

Fully adjusted models for: BMI, alcohol consumption, educational level and smoking. **p*-value < 0.05.

Supplementary Table 2. Men linear regression coefficient estimates with 95% confidence intervals for the epigenetic age and pace of aging.

	Levels	HorvathAA	HannumAA	PhenoAA	GrimaAgeAA	DunedinPoAm	DunedinPACE
**Current job classification:**							
NS-SEC	Semi-routine, routine worked/LT unemployed	Ref.	Ref.	Ref.	Ref.	Ref.	Ref.
	Lower supervisory and technical	1.824<br>(–0.23, 3.878)	0.949<br>(–0.718, 2.617)	1.169<br>(–1.351, 3.69)	0.138<br>(–1.417, 1.693)	–0.001<br>(–0.029, 0.028)	0.003<br>(–0.06, 0.066)
	Small employers and own account	0.883<br>(–0.958, 2.724)	0.032<br>(–1.463, 1.527)	0.787<br>(–1.472, 3.046)	–0.459<br>(–1.852, 0.935)	0.002<br>(–0.024, 0.028)	–0.031<br>(–0.087, 0.026)
	Intermediate	0.36<br>(–1.909, 2.63)	0.006<br>(–1.837, 1.848)	0.296<br>(–2.489, 3.081)	–0.929<br>(–2.647, 0.789)	–0.002<br>(–0.033, 0.03)	–0.015<br>(–0.084, 0.055)
	Management and professional	0.927<br>(–0.763, 2.618)	0.613<br>(–0.759, 1.986)	0.164<br>(–1.911, 2.239)	–0.576<br>(–1.856, 0.705)	–0.002<br>(–0.025, 0.022)	–0.057<br>(–0.109, –0.005)
Job sector	Other type of organization	Ref.	Ref.	Ref.	Ref.	Ref.	Ref.
	Private firm or business, a limited company	–1.098<br>(–2.452, 0.255)	–1.824<br>(–2.908, –0.741)	–3.251<br>(–4.866, –1.635)	–0.568<br>(–1.585, 0.449)	–0.017<br>(–0.036, 0.002)	–0.053<br>(–0.096, –0.01)
**Job stability:**							
Status contract	A permanent job	Ref.	Ref.	Ref.	Ref.	Ref.	Ref.
	Not permanent job	–0.112<br>(–2.761, 2.538)	–0.023<br>(–2.169, 2.123)	–0.556<br>(–3.792, 2.68)	–0.48<br>(–2.479, 1.52)	0.014<br>(–0.023, 0.05)	0.002<br>(–0.08, 0.083)

Status job	Paid employment(ft/pt)	Ref.	Ref.	Ref.	Ref.	Ref.	Ref.
	Self employed	–0.086 (–1.461, 1.289)	–0.456 (–1.551, 0.639)	0.494 (–1.181, 2.17)	–0.48 (–1.499, 0.539)	0.005 (–0.014, 0.024)	0 (–0.043, 0.042)
	Unemployed	0.418 (–2.587, 3.424)	1.155 (–1.239, 3.549)	4.694 (1.032, 8.356)*	2.112 (–0.116, 4.339)	0.067 (0.026, 0.108)*	0.122 (0.029, 0.215)*
Job security	Unlikely to lose their job	Ref.	Ref.	Ref.	Ref.	Ref.	Ref.
	Likely to lose their job	–0.5 (–2.696, 1.697)	1.118 (–0.675, 2.91)	0.896 (–1.817, 3.61)	1.063 (–0.558, 2.685)	0.028 (–0.002, 0.058)	0.045 (–0.026, 0.115)
Pay type	Salaried	Ref.	Ref.	Ref.	Ref.	Ref.	Ref.
	Paid by the hour	1.086 (–0.358, 2.53)	0.022 (–1.161, 1.206)	0.583 (–1.208, 2.375)	1.571 (0.507, 2.634)*	0 (–0.02, 0.02)	0.014 (–0.033, 0.061)
Has second job	No	Ref.	Ref.	Ref.	Ref.	Ref.	Ref.
	Yes	–0.498 (–2.446, 1.45)	–0.123 (–1.68, 1.435)	0.273 (–2.132, 2.678)	–0.874 (–2.328, 0.58)	–0.006 (–0.034, 0.021)	–0.004 (–0.066, 0.057)
**Job Schedule:**							
Working time	During the day	Ref.	Ref.	Ref.	Ref.	Ref.	Ref.
	Night	–1.086 (–3.886, 1.713)	–0.869 (–3.136, 1.399)	–0.524 (–3.948, 2.899)	0.881 (–1.233, 2.994)	0.016 (–0.023, 0.055)	0.047 (–0.039, 0.133)
	Rotating shifts	0.056 (–1.264, 1.376)	–0.173 (–1.242, 0.896)	–0.215 (–1.829, 1.399)	0.073 (–0.923, 1.07)	0.004 (–0.014, 0.022)	0.018 (–0.023, 0.059)
Working weekends	No weekend working	Ref.	Ref.	Ref.	Ref.	Ref.	Ref.
	Yes – some weekends	–0.187 (–1.408, 1.034)	–0.463 (–1.453, 0.526)	–0.959 (–2.45, 0.532)	–0.953 (–1.867, –0.04)	–0.01 (–0.026, 0.007)	–0.022 (–0.06, 0.015)
	Yes – most/every weekend	–0.864 (–2.317, 0.588)	–0.538 (–1.716, 0.639)	–0.32 (–2.094, 1.454)	0.078 (–1.01, 1.165)	–0.001 (–0.021, 0.019)	–0.023 (–0.068, 0.022)
Working hours	Less than 40 hours	Ref.	Ref.	Ref.	Ref.	Ref.	Ref.
	40 hours	–0.42 (–2.011, 1.171)	–0.943 (–2.228, 0.342)	–0.19 (–2.138, 1.759)	0.044 (–1.144, 1.233)	–0.006 (–0.028, 0.016)	–0.062 (–0.112, –0.011)
	More than 40 hours	–0.915 (–2.706, 0.877)	–1.621 (–3.068, –0.174)*	–2.238 (–4.432, –0.045)*	–0.939 (–2.278, 0.399)	–0.019 (–0.044, 0.006)	–0.054 (–0.111, 0.002)
**Autonomy and influence at work:**							
Managerial duties	Not manager/supervisor	Ref.	Ref.	Ref.	Ref.	Ref.	Ref.
	Foreman/supervisor	1.933 (0.163, 3.702)	0.972 (–0.496, 2.44)	1.505 (–0.703, 3.713)	0.471 (–0.875, 1.818)	0.009 (–0.016, 0.034)	0.015 (–0.043, 0.072)
	Manager	–0.644 (–2.082, 0.795)	–0.014 (–1.207, 1.179)	–0.555 (–2.351, 1.24)	–0.301 (–1.395, 0.794)	–0.007 (–0.028, 0.013)	–0.033 (–0.079, 0.014)
Job autonomy	(15, 20) High	Ref.	Ref.	Ref.	Ref.	Ref.	Ref.
	(10, 15) Medium	0.043 (–2.244, 2.329)	–0.878 (–2.728, 0.972)	–1.639 (–4.427, 1.148)	–1.295 (–3.015, 0.425)	–0.022 (–0.054, 0.009)	–0.088 (–0.157, –0.018)
	(0, 10) Low	-0.296 (-2.291, 1.7)	-0.458 (-2.073, 1.156)	–1.163 (–3.596, 1.27)	–0.854 (–2.355, 0.647)	0.003 (–0.024, 0.031)	–0.04 (–0.101, 0.021)
**Occupational physical activity:**							
OPA	Not very active/Not at all	Ref.	Ref.	Ref.	Ref.	Ref.	Ref.
	Fairly Active	–0.239 (–1.484, 1.006)	–0.44 (–1.448, 0.567)	–0.408 (–1.926, 1.11)	0.009 (–0.931, 0.949)	0.007 (–0.01, 0.024)	0.006 (–0.033, 0.044)
	Very Active	–0.448 (–1.954, 1.058)	–0.225 (–1.444, 0.993)	–1.067 (–2.903, 0.769)	0.306 (–0.831, 1.443)	0.002 (–0.019, 0.023)	0.007 (–0.04, 0.054)
**Feelings regarding the job:**							
Job satisfaction	Neither Satisfied/ Dissatisfied	Ref.	Ref.	Ref.	Ref.	Ref.	Ref.
	Dissatisfied	–2.062 (–4.86, 0.735)	0.123 (–2.151, 2.396)	0.653 (–2.777, 4.083)	–0.477 (–2.598, 1.643)	0.011 (–0.028, 0.05)	–0.01 (–0.096, 0.077)

	Satisfied	–1.604 (–4.152, 0.944)	–0.295 (–2.365, 1.776)	0.117 (–3.007, 3.241)	–0.317 (–2.248, 1.615)	0.006 (–0.03, 0.041)	–0.007 (–0.086, 0.072)
Job feelings	[6, 8) Positive	Ref.	Ref.	Ref.	Ref.	Ref.	Ref.
	(8, 11) Average	–0.419 (–1.708, 0.871)	–0.776 (–1.817, 0.266)	–1.5 (–3.065, 0.066)	–0.715 (–1.688, 0.258)	–0.026 (–0.044, –0.009)*	–0.045 (–0.084, –0.005)
	(11, 30) Negative	–0.851 (–2.185, 0.483)	–0.757 (–1.835, 0.321)	–1.409 (–3.029, 0.211)	–0.199 (–1.205, 0.808)	–0.006 (–0.024, 0.012)	–0.009 (–0.05, 0.032)

Fully adjusted models for: BMI, alcohol consumption, educational level and smoking. *p–value < 0.05.

Supplementary Table 3. Women linear regression coefficient estimates with 95% confidence interval for the epigenetic age and pace of aging.

	Levels	HorvathAA	HannumAA	PhenoAA	GrimaAgeAA	DunedinPoAm	DunedinPACE
**Current job classification:**							
NS-SEC	Semi-routine, routine worked/LT unemployed	Ref.	Ref.	Ref.	Ref.	Ref.	Ref.
	Lower supervisory and technical	–2.781 (–5.505, –0.057)	–0.095 (–2.12, 1.93)	–3.119 (–6.506, 0.267)	0.738 (–1.23, 2.705)	–0.003 (–0.04, 0.034)	–0.01 (–0.08, 0.06)
	Small employers and own account	0.965 (–1.005, 2.936)	–0.324 (–1.789, 1.14)	–0.291 (–2.74, 2.159)	–0.512 (–1.935, 0.911)	0.002 (–0.024, 0.029)	–0.043 (–0.094, 0.007)
	Intermediate	–0.284 (–1.83, 1.262)	–0.92 (–2.069, 0.229)	–1.27 (–3.192, 0.653)	0.618 (–0.499, 1.734)	–0.012 (–0.033, 0.009)	–0.014 (–0.054, 0.025)
	Management and professional	0.448 (–1.015, 1.911)	0.162 (–0.926, 1.25)	–0.564 (–2.383, 1.256)	–0.108 (–1.164, 0.949)	–0.023 (–0.042, –0.003)	–0.036 (–0.074, 0.001)
Job sector	Other type of organization	Ref.	Ref.	Ref.	Ref.	Ref.	Ref.
	Private firm or business, a limited company	0.523 (–0.664, 1.709)	0.328 (–0.531, 1.188)	0.274 (–1.153, 1.701)	0.024 (–0.809, 0.857)	0.005 (–0.011, 0.02)	0.008 (–0.022, 0.037)
**Job stability:**							
Status contract	A permanent job	Ref.	Ref.	Ref.	Ref.	Ref.	Ref.
	Not permanent job	0.476 (–1.922, 2.874)	1.155 (–0.603, 2.913)	0.009 (–2.939, 2.956)	1.149 (–0.561, 2.86)	0 (–0.032, 0.032)	0.023 (–0.038, 0.084)
Status job	Paid employment (ft/pt)	Ref.	Ref.	Ref.	Ref.	Ref.	Ref.
	Self employed	0.683 (–0.889, 2.255)	–0.414 (–1.578, 0.75)	–0.081 (–2.03, 1.868)	–0.781 (–1.891, 0.328)	0.005 (–0.016, 0.026)	–0.019 (–0.059, 0.02)
	Unemployed	0.274 (–2.165, 2.712)	1.797 (–0.009, 3.603)	2.478 (–0.546, 5.502)	1.097 (–0.625, 2.819)	0.017 (–0.016, 0.05)	0.001 (–0.061, 0.062)
Job security	Unlikely to lose their job	Ref.	Ref.	Ref.	Ref.	Ref.	Ref.
	Likely to lose their job	1.204 (–0.875, 3.282)	0.373 (–1.152, 1.898)	2.677 (0.183, 5.171)	0.513 (–0.96, 1.986)	–0.003 (–0.031, 0.024)	0.02 (–0.032, 0.072)
Pay type	Salaried	Ref.	Ref.	Ref.	Ref.	Ref.	Ref.
	paid by the hour	0.422 (–0.796, 1.64)	0.791 (–0.086, 1.668)	1.353 (–0.102, 2.807)	0.016 (–0.839, 0.87)	0.009 (–0.007, 0.025)	0.019 (–0.011, 0.049)
Has second job	No	Ref.	Ref.	Ref.	Ref.	Ref.	Ref.
	Yes	–1.329 (–3.108, 0.45)	0.294 (–1.037, 1.624)	0.656 (–1.563, 2.876)	1.443 (0.186, 2.699)*	0.026 (0.002, 0.05)*	0.023 (–0.022, 0.067)
**Job schedule:**							
Working time	During the day	Ref.	Ref.	Ref.	Ref.	Ref.	Ref.
	Night	0.92 (–1.829, 3.669)	2.223 (0.221, 4.224)*	4.445 (1.104, 7.786)*	3.302 (1.374, 5.229)*	0.067 (0.032, 0.103)*	0.068 (–0.001, 0.138)
	Rotating shifts	0.307 (–0.913, 1.527)	0.651 (–0.237, 1.539)	0.653 (–0.829, 2.136)	–0.265 (–1.121, 0.59)	0.006 (–0.01, 0.022)	–0.012 (–0.043, 0.019)
Working weekends	No weekend working	Ref.	Ref.	Ref.	Ref.	Ref.	Ref.

	Yes – some weekends	0.861 (–0.29, 2.013)	0.48 (–0.37, 1.33)	1.021 (–0.398, 2.441)	–0.369 (–1.197, 0.459)	0.003 (–0.013, 0.018)	0.002 (–0.027, 0.032)
	Yes – most/every weekend	1.223 (–0.235, 2.681)	0.16 (–0.916, 1.237)	0.681 (–1.115, 2.478)	0.025 (–1.023, 1.073)	–0.004 (–0.024, 0.016)	0.005 (–0.032, 0.043)
Working hours	Less than 40 hours	Ref.	Ref.	Ref.	Ref.	Ref.	Ref.
	40 hours	0.561 (–1.746, 2.868)	0.815 (–0.854, 2.483)	1.559 (–1.211, 4.33)	0.914 (–0.7, 2.528)	0.031 (0.001, 0.061)*	0.026 (–0.031, 0.084)
	More than 40 hours	1.838 (–1.988, 5.663)	1.174 (–1.592, 3.94)	0.771 (–3.822, 5.365)	1.247 (–1.43, 3.924)	0.014 (–0.036, 0.063)	–0.021 (–0.116, 0.074)
**Autonomy and influence at work:**							
Managerial duties	Not manager/supervisor	Ref.	Ref.	Ref.	Ref.	Ref.	Ref.
	Foreman/supervisor	–0.918 (–2.579, 0.743)	0.123 (–1.082, 1.327)	0.091 (–1.912, 2.094)	0.138 (–1.03, 1.305)	0.012 (–0.01, 0.034)	–0.006 (–0.048, 0.035)
	Manager	0.578 (–1.008, 2.164)	0.631 (–0.519, 1.781)	–0.353 (–2.265, 1.56)	–0.443 (1.557, 0.672)	–0.006 (–0.026, 0.015)	–0.008 (–0.047, 0.032)
**Job autonomy:**							
	(15, 20) High	Ref.	Ref.	Ref.	Ref.	Ref.	Ref.
	(10, 15) Medium	–0.689 (–2.513, 1.134)	–0.473 (–1.811, 0.865)	–2.321 (–4.546, –0.096)	–0.858 (–2.157, 0.44)	–0.019 (–0.044, 0.005)	–0.071 (–0.117, –0.025)
	(0, 10) Low	–0.102 (–1.808, 1.603)	0.178 (–1.074, 1.429)	–0.974 (–3.054, 1.106)	–0.065 (–1.28, 1.149)	–0.011 (–0.033, 0.012)	–0.045 (–0.088, –0.003)
**Occupational physical activity:**							
OPA	Not very active/ Not at all	Ref.	Ref.	Ref.	Ref.	Ref.	Ref.
	Fairly Active	–0.18 (–1.321, 0.961)	0.353 (–0.487, 1.194)	0.233 (–1.163, 1.629)	0.602 (–0.214, 1.418)	0.014 (–0.001, 0.029)	0.03 (0.001, 0.058)*
	Very Active	0.954 (–0.59, 2.498)	0.531 (–0.606, 1.669)	2.046 (0.157, 3.936)*	0.39 (–0.715, 1.495)	0.024 (0.003, 0.044)*	0.056 (0.017, 0.095)*
**Feelings regarding the job:**							
Job satisfaction	Neither Satisfied/Dissatisfied	Ref.	Ref.	Ref.	Ref.	Ref.	Ref.
	Dissatisfied	–0.468 (–3.071, 2.135)	–0.378 (–2.299, 1.542)	–3.865 (–7.024, –0.706)*	1.299 (–0.564, 3.162)	–0.01 (–0.045, 0.025)	–0.028 (–0.094, 0.038)
	Satisfied	0.695 (–1.427, 2.817)	–0.35 (–1.916, 1.215)	–0.758 (–3.333, 1.818)	0.857 (–0.661, 2.376)	–0.018 (–0.046, 0.01)	–0.047 (–0.101, 0.007)
Job feelings	(6, 8) Positive	Ref.	Ref.	Ref.	Ref.	Ref.	Ref.
	(8, 11) Average	0.242 (–1.082, 1.565)	0.207 (–0.766, 1.181)	0.221 (–1.406, 1.849)	0.002 (–0.946, 0.949)	–0.004 (–0.022, 0.014)	–0.008 (–0.042, 0.026)
	(11, 30) Negative	–0.163 (–1.366, 1.04)	0.067 (–0.818, 0.952)	–0.052 (–1.532, 1.428)	–0.141 (1.002, 0.721)	–0.008 (–0.024, 0.008)	0.008 (–0.023, 0.038)

Fully adjusted models for: BMI, alcohol consumption, educational level and smoking. *p–value < 0.05.
